# Base editors: a powerful tool for generating animal models of human diseases

**DOI:** 10.15698/cst2018.10.156

**Published:** 2018-09-28

**Authors:** Zongyang Lu, Xingxu Huang

**Affiliations:** 1 School of Life Science and Technology, ShanghaiTech University, 100 Haike Rd., Pudong New Area, Shanghai 201210, China.; 2 University of Chinese Academy of Sciences, 100049 Beijing, China.; 3 CAS Center for Excellence in Molecular Cell Science, Shanghai Institute of Biochemistry and Cell Biology, Chinese Academy of Sciences; University of Chinese Academy of Sciences, 320 Yueyang Road, Shanghai 200031, China.

**Keywords:** base editors, disease models, genome engineering, mice, non-human primates

## Abstract

Myriads of genetic mutations, including base substitutions, deletions, and insertions as well as chromosome structural variations, have been detected in many human diseases. Although current combination of genomics and bioinformatics has contributed greatly to understanding the genetics of these disorders, it remains challenging to ensure the causal functions of each mutation, and then to further investigate the underlying mechanism and to develop therapeutic strategies. Animal models generated by genome engineering are the key to address these issues. In this review, we will first revisit the limitation of conventional gene editing tools and mouse models generated in the past. We will then introduce a novel tool, base editors (BEs), which present a new promising approach to establish pathogenically relevant animal models. Finally, we will discuss the application of BEs in non-human primates and share our perspectives on future development of base-editing techniques.

## BASE EDITORS: A NOVEL GENOME ENGINEERING TOOL

Genetically engineered mouse models of human diseases have long been used in understanding the underlying mechanisms of the clinically relevant genetic disorders. In the past, using gene-targeting technology to establish animal models is time-consuming and laborious. Recently developed genome editing tools, including ZFNs (zinc-finger nucleases), TALENs (transcription activator-like effector nucleases) and CRISPR (clustered regularly interspaced short palindromic repeat), facilitate the generation of mouse models. However, although very useful, ZFNs and TALENs still suffered from two limitations. First, ZF (zinc-finger) protein and TALE (transcription activator-like effector) protein need to be fused to a nuclease, such as fok1. Fok1 function as a dimmer, therefore, pairs of ZF or TALE proteins are essential but sometimes tough to generate. Second, ZFNs- and TALENs-based genome targeting requires ZFN or TALE assembly for each targeted sequence, which is much less convenient than CRISPR, which needs only a short sgRNA (single-guided RNA). For these reasons, CRISPR has been broadly used for gene function annotation. Nevertheless, CRISPR/Cas9-assisted homologous recombination works poorly in modeling clinically relevant point mutations. Methods to precisely and efficiently introduce base substitutions across the genome has been long awaited.

**Figure 1:**
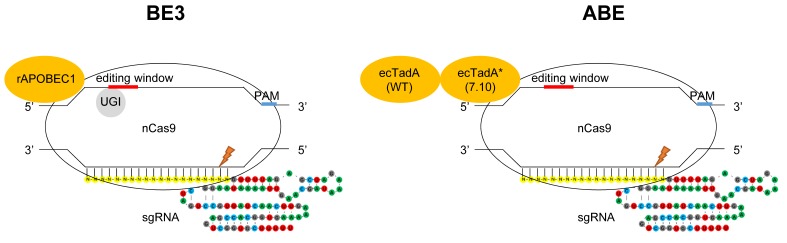
FIGURE 1: Targeted base substitutions without double strand break by cytidine- and adenine- deaminase. In these illustrations of base editing, deaminase proteins are shown in orange. The base-editing windows are highlighted in red, and the PAM sequences in blue on the DNA strand. nCas9 indicates Cas9 nickase harboring D10A mutation. Left: BE3 composed of rAPOBEC1, nCas9 and UGI (uracil glycosylase inhibitor). rAPOBEC1 indicates cytidine deaminase APOBEC1 derived from rat. Right: ABE composed of ecTadA (WT)-ecTadA* (7.10) fusion and nCas9. ecTadA (WT) indicates natural adenine deaminase *Escherichia coli* TadA. ecTadA* (7.10) indicates *Escherichia coli* TadA variant after protein evolution.

Novel single base editing tools named BEs (base editors) created by fusing the cytosine/adenine deaminase to the N-terminus of Cas9 nickase have been constructed to convert A to G or C to T at specific sites, without causing DSB (double-strand breaks) and with minimized off-target 1 (**Fig. 1**). Based on the fused deaminase enzyme, BEs can be categorized into CBE (cytosine base editor) and ABE (adenine base editor). For CBE, the rat APOBEC1 (rAPOBEC1) deaminase and sea lamprey AID ortholog (PmCDA1) deaminase are fused to Cas9 nickase, respectively 2,3. CBEs have been used in various species 4,5,6, including human embryos 7,8. To perform A to G conversion, ABE was engineered by fusing one natural adenine deaminase (ecTadA) and one adenine deaminase variant (ecTadA*) by protein evolution to the N-terminus of Cas9 nickase (**Fig. 1**). Both BE3 and ABE have an efficient editing window of approximately five nt, typically from C_4_/A_4_ to C_8_/A_8_ distal to PAM (**Fig. 1**).

BEs have accelerated the generation of clinically more relevant mouse models of human diseases caused by single-nucleotide substitutions 9. Base substitutions (be-tween C/G and T/A) account for ~54% known pathogenic human SNPs, suggesting the huge potential of BEs in modeling and correcting disease-associated mutations 1. In our studies, we chose androgen receptor (*Ar*) and homeobox D13 (*Hoxd13*) as targeted genes, and generated two mouse models, androgen insensitivity syndrome (AIS) and Syndactyly, by introducing A to G conversions at corresponding homologous pathogenic sites of patients through microinjection of mRNA and sgRNAs into zygotes. The Syndactyly model harboring modifications on *Hoxd13* was developed with germline transmission, demonstrating introduced mutations are heritable. Furthermore, considering different types of substitutions (including A to G and C to T) usually co-exist in same patients in clinic, we introduced A to G and C to T substitutions simultaneously *in vivo*. To avoid possible competition between BEs, we applied CBE-SaCas9 together with ABE-SpCas9, and successfully generated two types of substitutions at different targeted loci (*Hoxd13* and *Tyr*) simultaneously. As expected, the founder pups harbored two kinds of phenotypes, fused digits (*Hoxd13*) and white coat (*Tyr*), indicating the success in modeling complex diseases from multiple mutations. Furthermore, comprehensive off-target analysis by WGS and targeted deep sequencing analysis revealed no detectable off-target effects. Meanwhile, several groups also utilized BEs to generate premature stop codon to precisely disrupt endogenous genes 6,10, further demonstrating the versatility of BEs in modeling disease-relevant mutations. These studies pave the way to explore more than 30000 pathogenic SNPs across the genome

## NON-HUMAN PRIMATE MODELS TO RECAPITULATE HUMAN DISEASES

**Figure 2:**
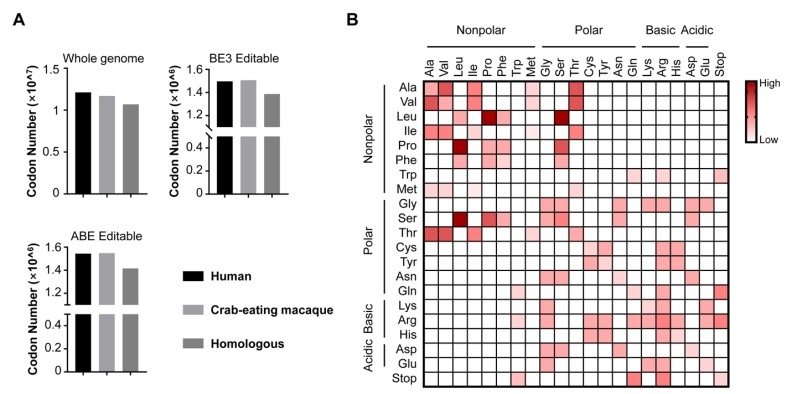
FIGURE 2: The analysis of editable codons for BEs. **(A) **The number of codons from NCBI database. Histograms in this panel show the number of codons of human and crab-eating macaque genomes as well as their homologous codons (Whole genome), and the number of codons from these two species editable with BE3 and ABE (BE3 Editable and ABE Editable, respectively). **(B)** Illustration of the repertoire of the amino acid conversions generated by BEs (BE3 and ABE). The amino acids on x axis and y axis are converted between each other. The theoretical abilities of amino acid conversions are displayed in red-white color scale.

As we known, non-human primates (NHPs) are considered as one of the best disease model organisms due to their similarities to humans in genetics, anatomy and physiology 11,12. Recent advances have proved the great promise of NHP models harboring genome modifications in biomedical research 13,14,15. We believe base editing in non-human primates will better mimic human diseases. By genome sequencing and comparison, NHPs displayed high similarity with humans on drug targets and homologous disease genes 16. We also analyzed the whole-genome sequences of human and crab-eating macaque from NCBI database, and found the number of codons (excluding start and stop codons) across whole genome of humans and crab-eating macaque are quite similar. There are about 1.2 × 10^7^ in humans and 1.1 × 10^7^ in crab-eating macaque, and most of them (1.0 × 10^7 ^codons) homologous (**Fig. 2A**). In addition, the editable codons for BE3 and ABE are more than 1.49 × 10^6^ in human and macaque, and most of them (~1.40 × 10^6^) are homologous too, indicating the high feasibility for modelling pathogenic mutations in NHPs using BEs (**Fig. 2A**).

## PROBLEMS AND PERSPECTIVES

Although BEs show their versatility in genome manipulation, two main concerns remain to be addressed. First, BEs are limited by PAM (protospacer-adjacent motif) and editing windows, therefore cannot cover the majority of sites of interest including many pathogenic sites. Increasing the genome-targeting scope of BEs is urgent. To address this concern, cytidine deaminase fusions with SaCas9 (PAM: NNGRRT) and Cpf1 (PAM: TTTN), respectively, have recently been developed 17,18. We previously developed a BE-PLUS tool with broadened editing window at targeted sites by using Suntag tail for recruiting 10 copies of APOBEC-UGI-GB1 19. A recently evolved Cas9 variant, xCas9, even provides more choices because of its broader PAM compatibility 20. Second, both A-to-G and C-to-T are base substitutions within pyrimidines or purines. However, base transversions (changes between pyrimidines and purines) account for a larger proportion of diseases. To date, it is hard to achieve base tranversions, because pyrimidines and purines have totally different molecular structures. As shown in **Fig. 2B**, the available amino acid substitutions generated by current BEs only account for 19% (80/420) of the missense mutations for the limitation of BEs that the mutated codons are only derived from pyrimidines/purines-to-pyrimidines/purines conversions. An efficient base transversion method will be highly desirable.

In conclusion, BEs have been successfully used to precisely edit the genomes of several organisms, including mammals, and have proven to be a powerful tool for modeling human diseases caused by single-base mutations. The applications of BEs in NHPs will provide us better models of human diseases and shed new light on our understanding of their pathophysiology.
